# Tuberculosis in an Urban Area in China: Differences between Urban Migrants and Local Residents

**DOI:** 10.1371/journal.pone.0051133

**Published:** 2012-11-30

**Authors:** Xin Shen, Zhen Xia, Xiangqun Li, Jie Wu, Lili Wang, Jing Li, Yuan Jiang, Juntao Guo, Jing Chen, Jianjun Hong, Zheng’an Yuan, Qichao Pan, Kathryn DeRiemer, Guomei Sun, Qian Gao, Jian Mei

**Affiliations:** 1 Key Laboratory of Medical Molecular Virology, Shanghai Medical College, Fudan University, Shanghai, People’s Republic of China; 2 Department of Tuberculosis Control, Shanghai Municipal Center for Disease Control and Prevention, Shanghai, People’s Republic of China; 3 Department of Tuberculosis Control, Songjiang District Center for Disease Control and Prevention, Shanghai, People’s Republic of China; 4 University of California, Davis, School of Medicine, Davis, California, United States of America; St. Petersburg Pasteur Institute, Russian Federation

## Abstract

**Background:**

The increase in urban migrants is one of major challenges for tuberculosis control in China. The different characteristics of tuberculosis cases between urban migrants and local residents in China have not been investigated before.

**Methodology/Principal Findings:**

We performed a retrospective study of all pulmonary tuberculosis patients reported in Songjiang district, Shanghai, to determine the demographic, clinical and microbiological characteristics of tuberculosis cases between urban migrants and local residents. We calculated the odds ratios (OR) and performed multivariate logistic regression to identify the characteristics that were independently associated with tuberculosis among urban migrants. A total of 1,348 pulmonary tuberculosis cases were reported during 2006–2008, among whom 440 (32.6%) were local residents and 908 (67.4%) were urban migrants. Urban migrant (38.9/100,000 population) had higher tuberculosis rates than local residents (27.8/100,000 population), and the rates among persons younger than age 35 years were 3 times higher among urban migrants than among local residents. Younger age (adjusted OR per additional year at risk = 0.92, 95% CI: 0.91–0.94, p<0.001), poor treatment outcome (adjusted OR = 4.12, 95% CI: 2.65–5.72, p<0.001), and lower frequency of any comorbidity at diagnosis (adjusted OR = 0.20, 95% CI: 0.13–0.26, p = 0.013) were significantly associated with tuberculosis patients among urban migrants. There were poor treatment outcomes among urban migrants, mainly from transfers to another jurisdiction (19.3% of all tuberculosis patients among urban migrants).

**Conclusions/Significance:**

A considerable proportion of tuberculosis cases in Songjiang district, China, during 2006–2008 occurred among urban migrants. Our findings highlight the need to develop and implement specific tuberculosis control strategies for urban migrants, such as more exhaustive case finding, improved case management and follow-up, and use of directly observed therapy (DOT).

## Introduction

Tuberculosis remains a serious public health problem worldwide, with 9.4 million new cases in 2009 [1]. The areas most affected by tuberculosis are low- to middle-income countries within the South-East Asia, African and Western Pacific regions. China has the second largest tuberculosis epidemic worldwide, behind only India [1]. The prevalence of tuberculosis in China is influenced by several factors, and the increase in urban migrants is one of the major challenges [2], [3].

China’s rapid urbanization process, led by the nationwide economic reforms, created large-scale domestic migrations. It is estimated that there will be 240 to 260 million urban migrants in China by 2030 [4]. Urban migrants leave their rural hometowns to cities to seek jobs. However, due to the household registration system (*hukou* system) in China [5], urban migrants and their families were not registered as official residents in the cities, and were not entitled to subsidized housing, education, social security or medical benefits [6], [7]. As a result, urban migrants had a lower social and economic level [8], a limited knowledge of tuberculosis [9], and a higher risk of tuberculosis than local residents [5]. In some urban areas in China, urban migrants accounted for at least one third of all tuberculosis cases since 2000 [10], [11]. Moreover, tuberculosis patients among urban migrants had low access to tuberculosis care [9] and poor treatment outcomes [10] and drug resistance rates [12].

Previous studies in industrialized countries reported that immigrants from high incidence areas were at a high risk of tuberculosis and contribute to the burden of tuberculosis in the host country [13], [14], [15]. In the United States and parts of Europe, the proportion of tuberculosis cases among immigrants exceeds 50%. Several characteristics of tuberculosis in immigrants have been identified, such as younger age [16], [17], [18], drug resistance [13], [17], [18], and poor treatment outcomes [17]. These characteristics help to design and target specific control strategies for the management of tuberculosis within immigrant populations. However, the characteristics of tuberculosis cases among urban migrants and local residents in China have not been previously investigated.

The objective of this study was to determine the demographic, clinical and microbiological characteristics of tuberculosis cases between urban migrants and local residents. The research results will be used for the design and implementation of specific tuberculosis control strategies among urban migrants.

**Table 1:** Number of pulmonary tuberculosis cases and tuberculosis case rates, among local residents and urban migrants in Songjiang district, Shanghai, 2006–2008.

Age group (years)	Urban migrants			Local residents			Rate ratio^b^
	Population (×100,000)	No. (%) of TB cases	TB rate(/100,000)^a^	Population (×100,000)	No. (%) of TB cases	TB rate(/100,000)^a^	
<18	2.8	26 (2.9)	9.1	2.1	6 (1.3)	2.9	3.1
18–34	13.2	629 (69.3)	47.7	3.9	62 (14.1)	15.8	3.0
35–59	6.8	226 (24.9)	33.0	6.5	157 (35.7)	24.1	1.4
≥60	0.4	27 (2.6)	59.5	3.3	215 (48.9)	65.1	0.9
Total	23.3	908 (100.0)	38.9	15.8	440 (100.0)	27.8	1.4

No. = number, TB = tuberculosis.

a TB rate = No. of pulmonary TB cases/population.

b Rate ratio = Pulmonary TB rate in urban migrants/Pulmonary TB rate in local residents.

## Methods

### Study Setting

Shanghai is a metropolis with approximately 17 million inhabitants in 2005, and one of the richest areas in China. We selected Songjiang district, one of 18 administrative districts in Shanghai, as our study area because it has a rapid rate of urbanization and the highest increase in the proportion of urban migrants among its population in Shanghai since the nationwide economic reforms started in 1980s. In 2006, Songjiang district had a population of approximately 943,700 persons, and 57.0% were local residents and 43.0% were urban migrants.

**Table 2:** Characteristics of pulmonary tuberculosis cases among urban migrants and local residents in Songjiang district, Shanghai, 2006–2008.

Characteristics	Urban migrants (n = 908)		Local residents (n = 440)		OR (95% CI)	p value
	No.	%	No.	%		
Sex						
Male	571	62.9	355	80.7	1.0	
Female	337	37.1	85	19.3	2.46 (1.86–3.28)	<0.0001
Age (years)						
Median (Mean)	28 (30.7)		58 (56.7)			<0.0001
Previous history of tuberculosis						
No	837	92.2	400	90.9	1.0	
Yes	71	7.8	40	9.1	0.85 (0.56–1.31)	0.426
Any comorbidity at diagnosis						
Absent	864	95.2	344	78.2	1.0	
Present	44	4.8	96	21.8	0.18 (0.12–0.27)	<0.0001
Cavitation on the initial chest radiograph^a^						
Absent	637	70.1	315	71.6	1.0	
Present	209	23.0	109	24.8	0.95 (0.72–1.25)	0.697
Sputum smear						
Negative	584	64.3	257	58.4	1.0	
Positive	324	35.7	183	41.6	0.78 (0.62–0.99)	0.036
Sputum culture						
Negative	569	62.7	292	66.4	1.0	
Positive	339	37.3	148	33.6	1.17 (0.92–1.50)	0.185
Treatment outcomes						
Success	688	75.8	406	92.3	1.0	
Poor outcome	220	24.2	34	7.7	3.82 (2.59–5.77)	<0.0001
Drug resistance pattern^b^						
Pan-susceptible^c^	207	78.4	97	79.5	1.0	
Any resistance^ed^	57	21.6	25	20.5	1.07 (0.61–1.90)	0.806
MDR^e^	20	7.6	9	7.4	1.04 (0.43–2.70)	0.923

No. = number, MDR = multi-drug resistance, OR = odds ratio; CI = confidence interval.

a For persons who had cavitation on the initial chest radiograph.

b For persons who had a drug susceptibility test result available.

c Defined as a strain with susceptibility to four first-line drugs (isoniazid, rifampin, streptomycin, and ethambutol).

d Defined as a strain with resistance to at least one of four first-line drugs (isoniazid, rifampin, streptomycin, and ethambutol).

e Defined as a strain with resistance to at least isoniazid and rifampin.

In Songjiang district, all suspected cases of pulmonary tuberculosis detected in a general hospital or a community health centre were referred to the district tuberculosis hospital, where the diagnosis was made by sputum smear, culture and chest radiography. According to the “Diagnostic criteria for pulmonary tuberculosis” issued by Ministry of Health, confirmed tuberculosis cases included individuals who were sputum smear positive, sputum culture positive, and individuals who have pulmonary lesions of tuberculosis that have been confirmed by pathological examination [19]. All patients with confirmed pulmonary tuberculosis must be reported to the Tuberculosis Registry at Songjiang District Center for Diseases Control and Prevention (CDC) through the mandatory notification system. In general, treatment for new pulmonary tuberculosis patients was 2 months of isoniazid, rifampicin, pyrazinamide, and ethambutol (or streptomycin), then 4 months of isoniazid and rifampicin (2HRZE(S)/4HR). Retreatment pulmonary TB patients received treatment using 2HRZES/1HRZE/5HRE or 2HRZES/6HRE. The phase of anti-tuberculosis therapy could be extended, depending on radiological and bacteriological data that were available and the clinical judgment of the treating physician. All of the pretreatment positive cultures in the district tuberculosis hospital were sent to the Tuberculosis Reference Laboratory at Shanghai Municipal CDC, where drug susceptibility testing and specimen identification were routinely performed [12], [20]. In this retrospective study, we analyzed all patients with confirmed pulmonary tuberculosis who lived in Songjiang district during 2006–2008. Patients who were infected with non-tuberculous mycobacteria (NTM) were excluded from the analysis.

### Statistical Methods

We used population data for 2006 to 2008 from the Songjiang District Population and Family Planning Committee to calculate tuberculosis case rates. Univariate and multivariate analyses were performed to determine the characteristics of the tuberculosis patients that were associated with urban migrants. Odd ratios (ORs) and 95% confidence intervals (CI) were calculated to measure the association between patient characteristics and the outcomes of interest at the univariate and multivariate level. All variables for which the association with outcome had a p-value <0.20 in the univariate analysis were considered for the multivariate logistic regression model. We performed forward stepwise model construction, and the log likelihood ratios of successive models were compared until the final, most parsimonious model was identified. A p-value of <0.05 was considered significant. Adequacy of the final model was assessed by the goodness-of-fit test. All of the analyses were completed using Stata statistical software (version 8.0 SE, Stata Corporation, College Station, Texas, USA).

Urban migrants are defined as individuals who leave their rural hometowns where they were registered after birth, and stay in the cities without local permanent residency. Local residents are defined as individuals who were registered in the cities after birth and stay in the cities with local permanent residency. Comorbidity was defined as a chronic illness, such as diabetes, cardiovascular disease, cancer, or chronic obstructive pulmonary disease (COPD), or a concurrent infectious disease such as hepatitis or pneumonia. If patients reported that they had a comorbidity or a doctor determined they had a comorbidity, information that a comorbidity existed should be noted in the TB case report, but precise information about the actual disease condition was not collected. Successful treatment outcome is defined as a cure and/or treatment completion, according to international definitions [21]. A poor treatment outcome is defined as death, default, treatment failure, transfer to another jurisdiction, and other outcomes (e.g., discontinued TB treatment on medical advice).

### Ethics Statement

The study was approved by the Ethical Review Committee at Shanghai Municipal CDC. Since this was a retrospective study and all patients’ information used in this study was routinely collected through the mandatory notification system, the requirement for informed consent was waived by the ethics committees listed above.

## Results

A total of 1,366 cases were reported to the Tuberculosis Registry at Songjiang CDC during January 1, 2006 to December 31, 2008. Of these, 18 were infected with NTM and were excluded from the analysis. Of the 1,348 pulmonary tuberculosis patients, 440 (32.6%) were local residents and 908 (67.4%) were urban migrants.

### Tuberculosis Rates during the Study Period

Tuberculosis rates per 100,000 persons among urban migrants were higher than among local residents for each age group except for the age group of ≥60 years (Table 1). The greatest disparities in case rates between urban migrants and local residents were among younger persons. Rates were 3 times higher among urban migrants aged <35 years relative to local residents. The disparity decreased with increasing age. Among persons in the age group of ≥60 years, the tuberculosis rate among urban migrants was similar to that among local residents. Persons aged ≥60 years accounted for most of the tuberculosis cases among local residents (47.3%), while among urban migrants, the largest proportion occurred in persons aged 18 to 34 years (69.5%).

### Characteristics of Patients

By univariate analysis, urban migrants were more likely than local residents to be female (OR = 2.46, 95% CI: 1.86–3.28, p<0.0001), to be younger (median age: 28 vs 58, p<0.0001), and to have a poor treatment outcome (OR = 3.82, 95% CI: 2.59–5.77, p<0.0001). However, the urban migrants were less likely to have any comorbidity of their tuberculosis disease at diagnosis (OR = 0.18, 95% CI: 0.12–0.27, p<0.0001), and to have a positive sputum smear (OR = 0.78, 95% CI: 0.62–0.99, p = 0.036). There were no statistically significant differences between local residents and urban migrants in the proportions that had a previous tuberculosis treatment, cavitation on the initial chest radiograph, sputum culture positivity, or drug resistance (Table 2).

Treatment outcomes of tuberculosis cases in Songjiang district was shown in table 3. A total of 1094 (81.2%) tuberculosis cases had suscessful treatment outcomes. The most common poor treatment outcome was transferred out (12.9%), followed by death (2.6%), treatment default (1.6%), treatment failure (1.5%), and other outcomes (0.5%). Compared with local residents, tuberculosis cases among urban migrants were less likely to have a treatment success, death and other outcomes, but were more likely to transfer out to another jurisdiction, to have a treatment failure or to default from therapy (Table 3).

**Table 3:** Treatment outcomes of pulmonary tuberculosis among local residents and urban migrants in Songjiang district, Shanghai, 2006–2008.

Treatment outcome	Total	Urban migrants	Local residents	Urban migrants vs Local residents	
	No. (%)	No. (%)	No. (%)	OR (95% CI)	p value
Cure/treatment completion	1094 (81.2)	688 (75.8)	406 (92.3)	1.0	
Transferred out	175 (12.9)	175 (19.3)	0 (0.0)	103.96 (18.23–4138.63)	<0.001
Treatment failure	16 (1.2)	14 (1.5)	2 (0.4)	4.13 (0.94–37.59)	0.064
Death	35 (2.6)	10 (1.1)	25 (5.7)	0.24 (0.10–0.52)	<0.001
Treatment default	21 (1.6)	21 (2.3)	0 (0.0)	13.00 (2.08–537.53)	<0.001
Other^a^	7 (0.5)	0 (0.0)	7 (1.6)	0.07 (0.001–0.55)	0.002
Total	1348 (100.0)	908 (100.0)	440 (100.0)	–	–

No. = number, OR = odds ratio; CI = confidence interval.

a Includes patients who discontinued treatment on medical advice.

The most common comorbidities that were observed in our study population included diabetes mellitus (51 cases), hepatitis B virus infection (12 cases), chronic obstructive pulmonary disease (9 cases), pneumonia (6 cases), and cardiovascular disease (5 cases, Table 4). The proportions of tuberculosis cases with any comorbidity were lower among urban migrants than among local residents for all cases and for age groups of 18–34 years, 35–59 years and ≥60 years. Alhough the proportion of tuberculosis cases with any comorbidity was higher among urban migrants than among local residents for the age group of <18 years, the number of cases in this age group was small. Non-infectious chronic diseases (diabetes mellitus, COPD, and cardiovascular diseases) and pneumonia were more common among local residents. However, HBV infection was more common among urban migrants than among local residents. Diabetes mellitus was the most common comorbidity among both local residents and urban migrants. Infection with hepatitis B virus (HBV) was the second most common comorbidity among urban migrants (Table 4).

**Table 4:** Distribution of comorbidities of pulmonary tuberculosis patients stratified by age group in Songjiang district, Shanghai, 2006–2008.

Age group (years)	Total	Diabetes mellitus	HBV infection	COPD	Pneumonia	Cardiovascular diseases	Others
	No. (%)	No. (%)	No. (%)	No. (%)	No. (%)	No. (%)	No. (%)
<18							
Urban migrants (n = 26)	2 (7.7)		1 (3.8)		1 (3.8)		
Local residents (n = 6)							
Subtotal (n = 32)	2 (6.2)		1 (3.1)		1 (3.1)		
18–34							
Urban migrants (n = 629)	5 (0.8)	2 (0.3)	2 (0.3)		1 (0.2)		
Local residents (n = 62)	2 (3.2)				1 (1.6)		1 (1.6)
Subtotal (n = 691)	7 (1.0)	2 (0.3)	2 (0.3)		2 (0.3)		1 (0.1)
35–59							
Urban migrants (n = 226)	30 (13.3)	12 (5.3)	6 (2.7)		1 (0.4)	1 (0.4)	10 (4.4)
Local residents (n = 157)	23 (14.6)	17 (10.8)			1 (0.6)		5 (3.2)
Subtotal (n = 383)	53 (13.8)	29 (7.6)	6 (1.6)		2 (0.5)	1 (0.3)	15 (3.9)
≥60							
Urban migrants (n = 27)	7 (25.9)	4 (14.8)		1 (3.7)			2 (7.4)
Local residents (n = 215)	75 (34.9)	16 (7.4)	3 (1.4)	8 (3.7)	1 (0.5)	4 (1.9)	39 (18.1)
Subtotal (n = 242)	82 (33.9)	20 (8.3)	3 (1.2)	9 (3.7)	1 (0.4)	4 (1.6)	41 (16.9)
All age group							
Urban migrants (n = 908)	44 (4.8)	18 (2.0)	9 (1.0)	1 (0.1)	3 (0.3)	1 (0.1)	12 (1.3)
Local residents (n = 440)	96 (21.8)	33 (7.5)	3 (0.7)	8 (1.8)	3 (0.7)	4 (0.9)	45 (10.2)
Total (n = 1348)	140 (10.4)	51 (3.8)	12 (0.9)	9 (0.7)	6 (0.4)	5 (0.4)	57 (4.2)

HBV = Hepatitis B virus, COPD = chronic obstructive pulmonary diseases.

### Multivariate Logistic Regression Analysis

Based on the multivariate analysis, the following variables were independently associated with tuberculosis among urban migrants compared to local residents: younger age (adjusted OR per additional year at risk = 0.92, 95% CI: 0.91–0.94, p<0.001), poor treatment outcome (adjusted OR = 4.12, 95% CI: 2.65–5.72, p<0.001), and lower frequency of any comorbidity at diagnosis (adjusted OR = 0.20, 95% CI: 0.13–0.26, p = 0.013). Although female sex and sputum smear positivity at diagnosis were significant in the univariate analysis, they were not significant in the full multivariate model and therefore were dropped from the final model.

## Discussion

Urban migrants in Songjiang district accounted for two thirds of all tuberculosis cases in the study population and experienced higher rates of tuberculosis than do local residents. Additionally, some demographic, clinical and microbiological characteristics were independently associated with tuberculosis cases who were urban migrants compared to local residents. Our findings highlight the need to develop and implement innovative tuberculosis prevention and control strategies among communities of domestic urban migrants.

In our study, the tuberculosis rates among urban migrants were higher than the tuberculosis rates among local residents. However, the disparities in tuberculosis rates decreased with increasing age of tuberculosis cases. Higher tuberculosis rates in the younger age group among urban migrants than among local residents may reflect both the high prevalence of tuberculosis and the increased risk of progression from recent infection of *M. tuberculosis* to active disease. Most of the urban migrants are from rural areas in the western and central regions of China. According to the national survey, the prevalence of tuberculosis in the western and central regions of China were much higher than in the eastern region, including Shanghai [22]. Additionally, tuberculosis prevalence in rural areas in China was approximately twice as high as tuberculosis prevalence in urban areas [22]. Therefore, it is not surprising that the prevalence rate of active tuberculosis among urban migrants was higher than among local residents. Shanghai had the policy of routine physical examination for urban migrants, including chest radiographs, for urban migrants seeking work. Individuals without a physical examination and health certificate cannot be employed legally. Considering the high tuberculosis rate among urban migrants, Shanghai’s policy of physical examinations should continue to be implemented to detect active tuberculosis early and to prevent transmission, although the cost-effectiveness of this policy needs to be assessed.

Urban migrants had a lower social and economic level than urban residents. Urban migrants tended to live in unsanitary, usually overcrowded dormitories provided by their employers or in crowded accommodations shared with other workers [23]. They undertook physically demanding jobs such as manual labor, factory work and service work [24], and were paid a very low wages [25]. Under such circumstances in the cities, urban migrants could have increased risk of progression from recent infection of *M. tuberculosis* to active disease.

Identifying differences in the characteristics of tuberculosis patients between subgroups can guide tuberculosis control program officials to develop specific prevention strategies and to allocate resources for different subgroups. In our study, there were some significant differences between tuberculosis cases among urban migrants and tuberculosis cases among local resident. First, tuberculosis cases among urban migrants were significantly younger than local residents. This result is consistent with previous studies [16], [17], [18]. This age difference is logical because most of the urban migrants are young workers and come to the urban areas seeking work and better economic conditions.

Second, we found that the odds of having any comorbidity at the time of a tuberculosis diagnosis was lower among urban migrants. This finding can be explained by the different age distribution between local residents and urban migrants. Common comorbidities, especially diabetes mellitus, may be prevalent because of the increasing rates of diabetes [26] and continued high rates of tuberculosis in China [1]. Diabetes mellitus is an independent risk factor for the development of active tuberculosis [27]. A recent systematic review also found that diabetes is associated with an increased risk of failure and death during tuberculosis treatment, and is also associated with an increased risk of relapse of tuberculosis [28]. Thus, increased attention to treatment of tuberculosis in people with diabetes is needed. On the contrary, whether tuberculosis is a causal risk factor for diabetes remains unknown [27]. In our study, HBV infection was the second most common comorbidity among urban migrants. As China is a country with highest burden of HBV infection (especially in rural areas where the urban migrants originate) [29], and HBV infection is a risk factor for drug-induced liver injury (DILI) [30], which could considerably impact the outcomes of anti-TB treatment [31], studies are needed to determine whether baseline testing and clinical monitoring of HBV infection should be implemented in an effort to prevent DILI and improve the outcomes of anti-TB treatment.

Third, urban migrants had a significantly higher proportion of poor treatment outcomes. This finding was also reported in other cities in China [10]. In our study, a higher proportion of urban migrants who were tuberculosis cases transferred out to another jurisdiction (19.3%), contributing to poor treatment outcome among urban migrants. Treatment default (2.3% vs 0.0%) and treatment failure (1.5% vs 0.4%) were also more frequent among the urban migrants than among local residents (Table 3). Therefore, better communications between medical staff and tuberculosis program staff in different regions could help locate transferred patients and provide more complete follow-up of patients to ensure the use of directly observed therapy (DOT) and completion of therapy. Starting in 2009, a new policy on cross-regional management of tuberculosis cases was implemented in China. A recent study showed that this policy was generally recognized and used by tuberculosis program staff, and could meet the requirement for management of tuberculosis cases who were transferred out, although more specific and focused working measures were required [32]. Measures to reduce the proportion of patients who transferred out could be another way to improve treatment outcomes of tuberculosis cases among urban migrants. Our previous study showed that free anti-tuberculosis treatment, in combination with a subsidy for living expenses and transportation, could increase the proportion of tuberculosis cases who were willing to stay and complete anti-tuberculosis treatment in Shanghai, and significantly improved treatment outcomes [33].

There are several limitations in our study. First, we did not include certain demographic and economic variables – such as annual income, employment status, and education level – because rigorous documentation of these data was not available in medical records. Second, the prevalence of comorbidity among TB patients could be underestimated because not all the tuberculosis patients were screened for comorbidities (such as diabetes mellitus), and some comorbidity information was self-reported by some patients. However, preliminary data from another ongoing study evaluating the quality of tuberculosis reporting information in Shanghai showed that the proportion of tuberculosis patients who fail to report a comorbidity is low (<10%).

In conclusion, we found a considerable proportion of tuberculosis cases occurred among urban migrants in Songjiang district, Shanghai and that some of the characteristics of urban migrants were independently associated with tuberculosis relative to local resisdents. Our findings highlight the need to develop and implement specific tuberculosis control strategies for urban migrants, such as more exhaustive case finding, case management and follow-up and use of DOT for better treatment outcomes.
